# Individualized combination therapies based on whole-exome sequencing displayed significant clinical benefits in a glioblastoma patient with secondary osteosarcoma: case report and genetic characterization

**DOI:** 10.1186/s12883-022-02920-x

**Published:** 2022-10-21

**Authors:** Guo-zhong Yi, Tai-chen Zhu, Tian-shi Que, Zhi-yong Li, Guang-long Huang

**Affiliations:** 1 Department of Neurosurgery, Nanfang Hospital, Southern Medical University, Guangzhou Dadao Bei Street, Guangzhou, 510515 Guangdong People’s Republic of China; 2 Nanfang Hospital, Southern Medical University, Guangzhou, 510515 Guangdong People’s Republic of China

**Keywords:** Second malignant neoplasm, Osteosarcoma, Glioblastoma, Whole-exome sequencing, Combination therapy

## Abstract

**Background:**

The incidence of osteosarcoma as a secondary neoplasm in glioblastoma patient is extremely rare. The genetic characteristic still remains unclear until now.

**Case description:**

We reported a 47-year-old female patient with multiple intracranial disseminations and infiltrations (splenium of the corpus callosum and lateral ventricular wall) of a rapid progressive glioblastoma underwent occipital craniotomy and total resection of all the enhancing lesions. Whole-exome sequencing and pathological examination revealed glioblastoma, IDH1 wild type, PTEN deficient, TERT mutated, NF1mutated, MGMT unmethylated. After surgery, the patient received combined therapeutic regimen of TTFields (tumor-treating fields) plus pembrolizumab plus temozolomide and TTFields plus everolimus, which displayed significant clinical benefits. During the combined therapeutic course, an extremely rare secondary malignant neoplasm occurred, femur MR and pathological detection of biopsy tissue demonstrated osteosarcoma. The result of whole-exome sequencing revealed 7 germline mutated genes (EPAS1, SETD2, MSH3, BMPR1A, ERCC4, CDH1, AR). Bioinformatic analysis showed the two germline mutations (MSH3 and ERCC4) induced deficiency in the DNA repair machinery, which resulting in the accumulation of mutations and may generate neoantigens contributing to the development of a secondary osteosarcoma in this case.

**Conclusion:**

Individualized combination therapies based on whole-exome sequencing displayed significant clinical benefits in this case. Germline MSH3 and ERCC4 mutation may induce a secondary osteosarcoma in glioblastoma patients.

## Background

Glioblastoma (GBM) is the most common adult primary brain tumor that occurs in the central nervous system. The standard of care for newly diagnosed GBM involves surgical resection followed by temozolomide concurrent with radiation therapy and adjuvant temozolomide. Despite recent therapeutic advance such as tumor-treating fields (TTFields), the survival rate of patients with glioblastoma continues to be poor with a median survival of less than 2 years [1]. The incidence of a second malignant neoplasm (SMN) in GBM patients is extremely low. SMNs are most likely occurred in long-term survival GBM patients which received previous radiation and chemotherapy [2]. To our knowledge, osteosarcoma as a SMN in GBM patients is extremely rare and only five cases have been reported before [3–6]. All these five secondary osteosarcomas were occurred intracranially with a long latent period and considered to be radiation-induced. Herein, we report a unique case of non-radiation-induced osteosarcoma of the femur in a GBM patient within a short latent period, and we also performed whole-exome sequencing to reveal the genetic characterization for the first time.

## Case presentation

**Fig. 1:**
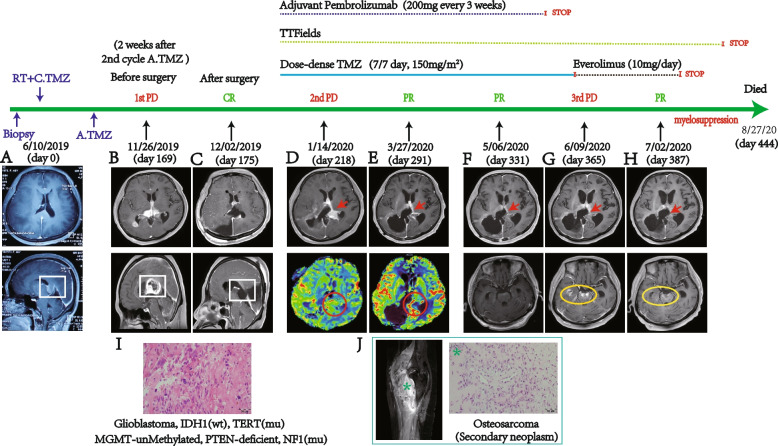
The timeline of treatments and typical MR, pathological images at the different stages. Brain MR images with post-gadolinium T1 before biopsy (**A**), before craniotomy surgery (**B**) and after craniotomy surgery (**C**) displayed rapid progress and intracranial metastasis of tumor, also demonstrated total resection of all enhancing tumor after craniotomy surgery. H&E staining demonstrated the classical appearance of a glioblastoma (**I**). Post-gadolinium T1 and contrast MR perfusion images of 6 weeks after surgery displayed rapid relapse at left lateral ventricular wall (**D**). After receiving combination therapies of dose-dense TMZ plus TTFields plus pembrolizumab for 10 weeks (**E**) and 16 weeks (**F**), MR images of post-gadolinium T1 and contrast MR perfusion displayed PR of relapsed tumor according to RANO criteria. Stop use of pembrolizumab for 4 weeks, several new enhancing lesions located at brainstem and temporal horn of right lateral ventricle were visible (**G**). Stop use of temozolomide, combination therapy of TTFields and everolimus for 4 weeks, brain MR images with post-gadolinium T1 demonstrated significant decreased in size of new metastasizing lesions (**H**). A secondary malignant neoplasm occurred during combined therapeutic course, femur MR and pathological detection of biopsy tissue demonstrated a secondary osteosarcoma (**J**). C. TMZ = concomitant Temozolomide; A. TMZ = adjuvant Temozolomide; mu = mutated; wt = wild type; CR = complete response; PR = partial response; PD = progressive disease

A 47-year-old female patient without familial tumor history was admitted to the hospital because of a 2-week history of visual disturbance with muscae volitantes symptom. The examination of visual acuity and field showed no abnormal. However, the brain MRI displayed multiple abnormal signals occurring, which located at right parietooccipital lobe with diffuse high signal intensity on T2-weight images and the lesion located at splenium of the corpus callosum with light enhancing. The patient underwent a biopsy operation on right parietooccipital lobe lesions and the pathological detection demonstrated diffused astrocytoma (Fig. 1A). Following, the patient received radiotherapy (comprising of 2-Gy daily fractions; day 1 to day 5 per week; total dose 60-Gy) and concomitant chemotherapy with temozolomide (75 mg/m^2^/day). Four weeks after the radio-chemotherapy, she also received adjuvant chemotherapy with temozolomide (150–200 mg/m^2^/day, 5/28 day per cycle).

After the first cycle of adjuvant Temozolomide (TMZ) chemotherapy, the patient presented to our institution with a 7-days history of progressively weakness. The neurological examination of the patient showed hypersomnia, hemiparesis of left limb. Compared with previous MRI, the MRI of this time in our institution displayed a significant enlargement of the lesion located at splenium of the corpus callosum with significant enhancement, the right parietooccipital lesion remain stable. New visible enhancing lesion was also detected at right lateral ventricular wall of occipital angle, which indicated possible multiple intracranial metastasis of tumor (Fig. 1B). We performed occipital craniotomy and conducted total resection of all the enhancing lesions with intraoperative neuro-navigation and electrophysiological monitoring (Fig. 1C). Whole-exome sequencing and pathological examination revealed glioblastoma, IDH1 wild type, PTEN deficient, TERT mutated, NF1mutated, MGMT unmethylated (Fig. 1I).

After surgery, the patient received combined therapeutic regimen of dose-dense adjuvant TMZ chemotherapy (150 mg/m^2^/day, 7/7 day per cycle) plus TTFields plus pembrolizumab (200 mg, every 3 weeks). No steroids were administered during treatment. Subsequent MRI (2 weeks after combination therapy) with post-gadolinium T1 and contrast perfusion revealed tumor rapid relapse at left lateral ventricular wall (Fig. 1D). Considering the time window of treatment works, the combination therapy continued. The follow-up MRI (10 weeks after combination therapy) displayed a progressive decrease in lesion size (Fig. 1E). Subsequent MRI (16 weeks after combination therapy) revealed partial response according to the RANO criteria (Fig. 1F). However, pembrolizumab was discontinued for the reasons of patient’s family. After stopping pembrolizumab for 4 weeks, several new enhancing lesions at brainstem and temporal horn of right lateral ventricle were visible (Fig. 1G). Oral mTOR inhibitor everolimus demonstrated significant disease stability/shrinkage in recurrent/progressive NF1-associated low grade glioma with a well-tolerated toxicity profile [7]. According to the NF1 mutated and PTEN deficient background of tumor, we stopped use of temozolomide and add oral everolimus into combined therapeutic schedule for salvage therapy. After receiving combination therapy of TTFields and everolimus for 4 weeks, the newly enhanced lesions shrank by over 90% (Fig. 1H). However, everolimus was ceased due to severe myelosuppression reaction (lymphocyte count: 0.28 × 10^9^/L, platelet count: 26 × 10^9^/L). Further, the myelosuppression was persistent and fail to remission. Almost 8 weeks later, the patient passed away due to severe lymphopenia and pulmonary infection.

In this case, an extremely rare secondary malignant neoplasm occurred during combined therapeutic course, femur MR and pathological detection of biopsy tissue demonstrated a secondary osteosarcoma (Fig. 1J). The secondary osteosarcoma remained stable during combination therapy. In order to reveal the potential genetic mechanism of secondary osteosarcoma occurrence, we analysis the result of whole-exome sequencing and found 8 germline gene variations, which consist of 7 germline genes (including EPAS1, SETD2, MSH3, BMPR1A, ERCC4, CDH1 and AR; shown in Table 1). All the combined therapeutical regimen described above were approved by the medical ethical committee and obtained informed consent of both the patient and patient’s relative.

**Table 1:** List of 8 germline gene variation sites identified in the glioblastoma

Gene	Chromosome	site	RSID	Gene type	Protein reference	Variant type
EPAS1	chr2	46,607,682	rs768469246	Heterozygote	p.A624D	Missense
SETD2	chr3	47,125,703	rs758229404	Heterozygote	p.N1856S	Missense
MSH3	chr5	79,950,708	NA	Heterozygote	p.A60_A62dup	Non-frame shift insertion
BMPR1A	chr10	88,679,172	NA	Heterozygote	p.K371T	Missense
ERCC4	chr16	14,015,937	rs187435008	Heterozygote	p.R86H	Missense
CDH1	chr16	68,867,343	rs142927667	Heterozygote	p.E864K	Missense
AR	chrX	66,765,158	rs3032358	Homozygote	p.Q79_Q8 0dupl	Non-frame shift insertion
AR	chrX	66,766,356	NA	Heterozygote	p.G473del	Non-frame shift deletion

*NA* Not accessible

## Discussion and conclusions

Glioblastoma is the most lethal primary central nervous system tumor with a median survival of less than 2 years. Despite therapeutic advances, glioblastomas present relapse almost without exception. In recurrent glioblastoma, median overall survival is an estimated 24 to 44 weeks [8]. Currently, the National Comprehensive Cancer Network (NCCN) has added TTFields to the list of recommended therapies for the treatment of glioblastoma. Though immunotherapy has demonstrated limited efficacy in patients with glioblastoma, PD-1 blockade in isolated case reports associated with mismatch repair deficiency showed clinical benefits [9]. More new therapies or combined therapied are needed for patients diagnosed with this type of cancer.

For glioblastoma, several clinical trials incorporating TTFields with combination therapies are ongoing, which consist of TTFields plus temozolomide plus pembrolizumab in newly diagnosed glioblastoma (2-THE-TOP, NCT03405792) [10]. In this case, the patient underwent biopsy operation, radiation and temozolomide therapy before admitting to our institution. We performed re-operation of gross total resection on the progressively enhancing tumor, molecular and pathological detection revealed glioblastoma, IDH1 wild type. Further, the patient received combined therapeutic regimen of TTFields plus temozolomide plus pembrolizumab. Though brain MRI of 6 weeks after surgery displayed glioblastoma relapse at tumor residual cavity again. After receiving combined therapeutic regimen, the relapsed tumor showed significant remission for almost 5 months. Until to stop pembrolizumab, tumor progressed at brainstem and temporal horn of right lateral ventricle. According to the NF1 mutated and PTEN deficient background of tumor, we stopped use of temozolomide and add oral mTOR inhibitor everolimus into combined therapeutic schedule for salvage therapy, the relapsed tumor displayed remission. However, all treatment discontinued because of severe myelosuppression reaction.

Secondary malignant neoplasm can occur in long-term survival GBM patients received previous radiation and chemotherapy [2]. To the best of our knowledge, osteosarcoma as a SMN in GBM patients is extremely rare and only five cases have been reported before [3–6]. All these five secondary osteosarcomas were occurred intracranially with a long latent period and considered to be radiation-induced. In this case, a secondary femur osteosarcoma occurred during the combined therapeutic course. Obviously, the secondary femur osteosarcoma was not induced by radiotherapy for tumor was not originating from irradiated field. Previous study has reported that the development of second primary cancers could point to underlying germline mutations that could include p53 (Li-Fraumeni syndrome), NF1 (neurofibromatosis type 1) or mismatch repair genes (Lynch syndrome) [11]. In order to reveal the potential genetic mechanism of secondary osteosarcoma occurrence, we analysis the result of whole-exome sequencing and found 8 germline gene variations, which consist of 7 germline genes (including EPAS1, SETD2, MSH3, BMPR1A, ERCC4, CDH1 and AR). Of note, among these mutated germline genes, both MSH3 and ERCC4 are DNA damage repair related genes and involved in maintenance of genome stability [12, 13]. And the result of germline mutation in MSH3 indicated the patient suffer constitutional mismatch repair (MMR) deficiency (CMMRD) or Lynch syndrome [14, 15]. We speculated that both of the two germline mutations (MSH3 and ERCC4) induce deficiency in the DNA repair machinery, which resulting in the accumulation of mutations and generate neoantigens contributing to the development of a secondary osteosarcoma in this case. However, this speculated mechanism need to be further proved in future studies.

In conclusion, we reported a unique case of secondary femur osteosarcoma in a GBM patient for the first time. We have also identified germline MSH3 and ERCC4 mutation may be the driver mutation of the secondary osteosarcoma. Moreover, although the final treatment outcome was frustrating, the individualized combination therapies of TTFields plus pembrolizumab plus temozolomide based on PTEN mutation and TTFields plus everolimus based on NF1 mutation displayed significant clinical benefits, which need further exploration in future clinical trials. Our report could serve as a helpful reference for clinicians and researchers.

## Data Availability

Not applicable.

## Abbreviations

GBM: Glioblastoma
TTFields: Tumor-treating fields
SMN: Second malignant neoplasm
TMZ: Temozolomide
C. TMZ: Concomitant Temozolomide
A. TMZ: Adjuvant Temozolomide
MU: Mutated
WT: Wild type
CR: Complete response
PR: Partial response
PD: Progressive disease
NCCN: National Comprehensive Cancer Network
NF1: Neurofibromatosis type 1
MMR: Mismatch repair
